# Spanish Version of the mHealth App Usability Questionnaire (S-MAUQ): Translation, Adaptation, and Validation Study

**DOI:** 10.2196/64787

**Published:** 2026-04-20

**Authors:** Antonio L Carrillo-León, Juan Falgueras-Cano, Esther Díaz-Mohedo

**Affiliations:** 1 Department of Languages and Computer Science, University of Málaga, C/ Doctor Ortiz Ramos s/n (Campus de Teatinos), Málaga, 29071, Spain, 34 951952390; 2 Department of Physiotherapy, University of Málaga, Málaga, Spain

**Keywords:** mHealth, usability questionnaires, usability evaluation, medical informatics, mobile health, app, mobile application, translation, adaptation, validation study, questionnaire-based evaluation, methodology, Spanish version, S-MAUQ, reliability, internal consistency, kappa statistic, digital health

## Abstract

**Background:**

In the current digital landscape, ensuring optimal usability is one of the most crucial factors determining the success of any mobile app. Questionnaire-based usability evaluations represent a highly prevalent methodology for this purpose. To date, questionnaires have been developed to assess the general system usability; however, there are hardly any questionnaires specifically designed to assess the usability of mobile health (mHealth) apps. The most widespread, the mHealth App Usability Questionnaire (MAUQ), has been developed in 4 versions according to the type of app (interactive or standalone) and the target user (patient or provider).

**Objective:**

The objective of this study was to translate and validate the English version of the MAUQ (standalone, for patients) into a Spanish version (S-MAUQ).

**Methods:**

The methodology used here follows that proposed by Sousa and Rojjanasrirat, which comprises 4 stages. The initial stage of the process entails a translation, harmonization, and adaptation procedure. The second and third entailed content validation (by 10 experts) and face validation (by 12 target users), respectively, which were conducted to evaluate the relevance and clarity of the questionnaire items. The item-level content validity index, scale content validity index (S-CVI), item-level face validity index, and scale face validity index (S-FVI), as well as the modified kappa statistic (κ) were used to evaluate interrater agreement among the raters, considering the probability of agreement by chance (Pc). The fourth and final stage of the process involved the assessment of the questionnaire’s reliability. A sample of 61 young adult participants installed an mHealth app (the Yazio app), used it, and responded to the S-MAUQ. The Cronbach α value for the entire questionnaire and its subscales were then calculated.

**Results:**

For the second stage, the S-CVI was initially 0.778. We removed items #14 and #15 from the Spanish version as they were unclear and not relevant. The S-CVI changed to 0.881. The third stage had an S-FVI of 0.927, indicating that the items are clear and straightforward for the nonexpert target user to understand. Furthermore, with each κ value >0.74, the validity of the instrument is supported. The fourth stage demonstrated the reliability of the S-MAUQ with a Cronbach α value of 0.87.

**Conclusions:**

The final version of the S-MAUQ met the validation criteria, demonstrating reliability and validity that are comparable with those of the original version. Consequently, the S-MAUQ is suitable for evaluating the usability of mHealth apps for young Spanish adults. Further research involving larger and more diverse samples is recommended.

## Introduction

mHealth is an acronym for mobile health, which is used to describe the practice of medicine and public health that is supported by mobile devices [1]. Such mobile devices encompass mobile phones, tablets, and wearable devices, including smart watches. mHealth apps can collect health information for practitioners, researchers, and patients alike and can monitor patients’ vital signs in real time. They facilitate direct care using mobile telemedicine and are used in the training of and collaboration with health workers [2].

The use of mHealth apps is increasing at a considerable rate [3], as evidenced by the growing number of users or patients [4]. Moreover, the emergence of innovative eHealth and mHealth technologies has the potential to significantly improve the cost-effectiveness of the health care system [5].

Presently, these health apps are typically developed either by researchers or, in the majority of cases, by commercial companies, without collaboration between these two groups. The lack of interaction between researchers and commercial developers in the field of health-related apps has resulted in a situation where commercially available apps have not been subjected to scientific validation and apps that have been developed from research projects are not commercially available [6].

It is imperative that mHealth apps are designed in a manner that ensures optimal *usability*. Such apps must be straightforward to use and capable of achieving their intended objectives in an efficient manner. Well-designed mHealth apps have demonstrated cost-effectiveness through the education and empowerment of patients as well as through improved treatment adherence. However, some reviews have highlighted the absence of criteria for assessing the validity of mHealth apps.

mHealth apps can be classified into two principal categories: those designed for patients and those designed for health care providers. *Patient-focused* apps are designed to assist with the management of health behaviors, whereas those oriented toward *health care providers* facilitate services such as prescriptions and education. Furthermore, mHealth apps can be classified into two additional categories: *interactive* apps, which facilitate communication with health care providers, and *standalone* apps, which include features such as reminders but lack provider interaction [7].

The optimal *usability* of apps is characterized by several key attributes, including efficiency, satisfaction, learnability, memorability, and low error rates [7,8]. However, the intrinsic constraints of mobile devices, notably their limited display area, can present obstacles to the attainment of these objectives [9].

The People at the Center of Mobile App usability model considers the user, task, and context of use to evaluate usability. This includes the cognitive load associated with tasks such as exercising while using the app, as discussed by Harrison et al [10]. To minimize errors, usability should be assessed using the following criteria: effectiveness, efficiency, satisfaction, learnability, memorability, and cognitive load [10].

A few usability scales have been evaluation to ascertain their relative importance in the context of the development of general software systems and mobile apps. For example, the Mobile App Rating Scale (MARS) [11], its user version (uMARS) [12], System Usability Scale [13,14], and Post-Study System Usability Questionnaire [15]. However, these scales are not appropriate for evaluating mHealth apps, as they were not developed with mHealth apps as a specific focus.

One of the most pivotal usability scales for assessing the usability of mHealth apps is the mHealth App Usability Questionnaire (MAUQ) [7]. This assesses the ease of use, interface, satisfaction, and usefulness of mHealth apps for end users.

The standalone for patient version of MAUQ has been translated, among other languages, into Malay, German, and Italian [16-18]. It is noteworthy that, while this research was being developed, another related study was published (a Spanish version of MAUQ standalone, for patient [19]) but adapted to breastfeeding support apps. However, it has too many differences from the original and some incorrect formulas, as will be addressed in the discussion section.

The objective of our study was to translate and validate the English version of the MAUQ (standalone, for patients) into a Spanish version (S-MAUQ) for use in future mHealth app research and development.

The importance of the translation into Spanish and its corresponding validation lies in two fundamental points: on the one hand, the large size of the Spanish-speaking community and, on the other, the cultural diversity of the type of user of these apps. Without this linguistic adaptation, these questionnaires would be out of reach for many users.

## Methods

### Overview of the Questionnaire

As previously stated, the MAUQ, developed and validated by Zhou and colleagues [7], is designed to assess the usability of mHealth apps among patients and health care providers. There are two versions of MAUQ, one for interactive apps and one for stand-alone apps. Each also addresses two of its potential users: patient or health care professional. We will work on MAUQ for standalone apps intended for patients. Always consider that the term “patient” refers to a person who uses an mHealth app to manage their health either for maintenance or improvement.

The questionnaire is comprised of 3 subscales or dimensions: ease of use (items #1 to #5), interface and satisfaction (items #6 to #12), and usefulness (items #13 to #18). Participants are required to indicate their level of agreement with each item on a 7-point Likert scale, with 1 representing strong disagreement and 7 representing strong agreement. The overall usability of the app is determined by calculating the total score and the mean score of all statements. A higher overall mean score is indicative of superior app usability. Conversely, a mean score <4 indicates suboptimal usability.

The original MAUQ (standalone, for patient version) exhibited robust internal consistency, with an overall Cronbach α value of 0.914. This indicates a high degree of correlation between the questionnaire items, indicating good reliability. The study was conducted in 2020 [7].

**Figure 1.**
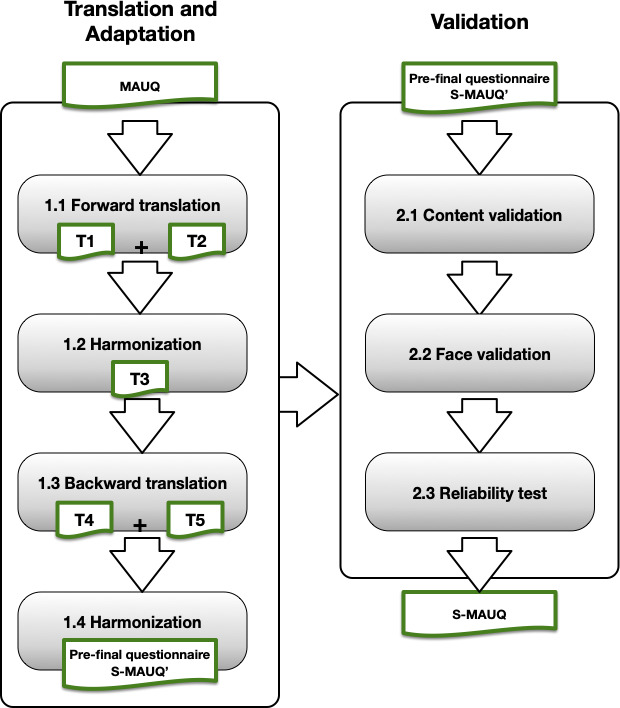
The flow of translation, cross-cultural adaptation, and validation process for the Spanish version of the mHealth App Usability Questionnaire (S-MAUQ).

The methodology used to translate and validate the original MAUQ (standalone, for patient version) into Spanish followed the approach proposed by Sousa and Rojjanasrirat [20], with the objective of ensuring equivalence between the original and translated versions. The process is comprised of several steps (illustrated in Figure 1), which can be grouped into the following 4 stages: translation and adaptation, content validation, face validation, and internal consistency reliability.

### Translation and Adaptation Process

The preliminary phase of the translation process entailed the participation of two certified translators, each with a specialized understanding of both Spanish culture and linguistics. These professionals engaged in a forward translation from English to Spanish. One translator had a background in computer science, while the other had a background in health sciences. This resulted in the generation of two distinct documents designated T1 and T2.

Subsequently, the documents (T1 and T2) underwent a harmonization process. A third translator, a Spanish language teacher with more than 5 years of teaching experience, was tasked with identifying any ambiguities; discrepancies; and differences in words, sentences, grammar, and meaning. This was accomplished through a comparison of T1 and T2 with the original MAUQ. Any discrepancies and ambiguities were discussed and resolved through a process of consensus decision-making involving the third translator, the first two translators, and two members of the research team. The process culminated in the production of an initial translated version of the MAUQ document (T3).

In the third phase of the process, the translated version of the MAUQ (T3) was provided to 2 independent native Spanish speakers who had acquired English as a second language. One individual (a computer science lecturer) was a content expert in the instrument’s construct, while the other had no prior knowledge of the instrument (an English language teacher). The 2 participants then produced independent back-translated versions of the MAUQ (T4 and T5).

Last, a committee was established comprising 3 researchers, the translators involved in the initial and concluding stages, and the original developers of the MAUQ (when necessary). The committee conducted a comparative analysis of the documents with the original MAUQ, engaging in discussions via email and web-based meetings. Any ambiguities or inconsistencies in cultural meaning and expression of words and sentences and the format of the answers were addressed and resolved through consensus among the committee members. This process resulted in the development of the prefinal Spanish questionnaire, designated as S-MAUQ’.

### Content Validation and Face Validation

Content validation is achieved by a panel of experts who assess the relevance and clarity of questionnaire items for measuring the usability of health-related mobile apps. Face validation is accomplished by a set of nonexperts or target users who specifically assess the clarity of the items.

#### Participants

For the content validation, a panel of 10 experts was formed. Our panel included 3 mobile app developers, 2 senior lecturers with a PhD in computer science, 2 usability experts, and 3 PhD senior lecturers and health-related practitioners.

For the face validation, 12 nonexpert participants were involved.

#### Recruitment

For the content validation, the 10 expert participants were carefully selected in order to adequately cover all areas of expertise involved. After being informed of the study’s objective, all participants signed the corresponding informed consent form.

For the face validation, users were randomly selected from an openly chosen sample. The inclusion criteria were as follows: (1) native Spanish speaker, (2) older than 18 years, and (3) familiar with smartphones. The exclusion criteria were being (1) health-related practitioners, (2) mobile app developers, or (3) computer science students or professionals.

Participants were asked to complete an anonymous web form that included the informed consent, gender, date of birth, and level of education.

#### Setting

The group of expert participants completed their evaluations remotely and sent them to us via email. The nonexpert participants completed their evaluation online via an anonymous web form.

#### Study Procedures

In the content validation stage, each expert is required to assign a score on a scale of 1 to 5 to each item. A score of 1 indicates that the item was not relevant or clear, while a score of 5 indicates that the item was highly relevant and clear. Subsequently, scores of 1 to 3 were recategorized as 0, indicating irrelevance or unclarity, while scores of 4 and 5 were recategorized as 1, indicating relevance or clarity. The final categorization was conducted with the objective of obtaining dichotomous values. In numerous other studies, the range extends from 1 to 4. In contrast, in our study, the range was from 1 to 5. However, the current quality standards are more demanding, as a rating of 3 was considered unsatisfactory. In addition, there were 3 instances of unsatisfactory performance, as opposed to only 2 instances of acceptable performance.

On the other hand, in the face validation, which was less demanding with the nonexpert evaluators, the scores ranged from 1 (indicating that the item was not understandable) to 4 (indicating that the item was very understandable). Scores of 1 and 2 were recategorized as 0 (not understandable), and scores of 3 and 4 were recategorized as 1 (understandable). This was less demanding because the chosen scores were taken directly without considering the middle 3 negative values as we did with the experts.

The calculations for the 2 types of evaluation were as described in the following statements.

If we have *N* as the number of raters or evaluators; *A_C_* as the number of expert raters who concur that the items are relevant, unambiguous, and intelligible; and *A*_*F*_ as the number of nonexpert raters who concur that the items are clear and comprehensible, the item-level content validity index (I-CVI) and the item-level face validity index (I-FVI) were calculated by dividing the propoortion of raters who agreed that an item was clear and relevant to the domain by the total number of raters.

In conclusion, it can be proposed that:

$I-CVI=\frac{A_{C}}{N}$ and $I-FVI=\frac{A_{F}}{N}$

Items with an I-CVI or I-FVI of at least 0.79 were relevant, unambiguous, and understandable to the target users, indicating their potential inclusion in the questionnaire [21,22].

The scale content validity index (S-CVI) and scale face validity index (S-FVI) can be calculated as the means of the corresponding I-CVI and I-FVI. This is accomplished by dividing the sum of each item by the total number of items [23].

Moreover, it is essential to determine the level of agreement among the 10 experts. This can be accomplished by calculating the modified kappa statistic [21,24,25], which can be expressed as follows:

$\kappa_{CVI}=\frac{\text{I-CVI}-P_{c}}{1-P_{c}}$ and $\kappa_{FVI}=\frac{\text{I-FVI}-P_{c}}{1-P_{c}}$

In this formula, *P_C_* represents the probability of chance occurrence. The initial determination of the probability of agreement by chance for each item was determined using the following formula (the formula for a binomial random variable) [21]:


Pc=(NA)⋅0.5N=N!A!(N−A)!⋅0.5N

This equation has its origins in the field of probability theory, specifically in the binomial distribution. The probability of obtaining precisely *A* successes in *N* independent Bernoulli trials (with the same rate *p*, in this case *1/2*) is given by the probability mass function (or discrete probability density function):

Pc=(NA)pk(1−p)N−A and for p=1/2 → Pc=(NA)0.5N

**Figure 2.**
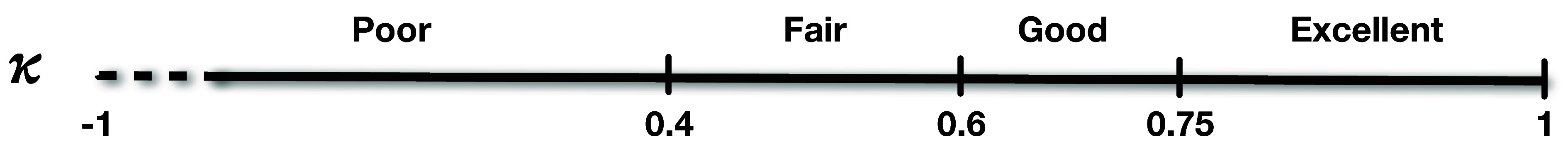
Interpretation criteria for the modified kappa statistic.

Graphically, the interpretation criteria for the modified kappa statistic, as outlined by Wynd et al [26] and Cicchetti and Sparrow [27], is shown in Figure 2.

### Reliability (Internal Consistency) Validation

#### Participants

For the internal consistency validation, the participants were selected from among students enrolled in the School of Engineering at the University of Malaga and the Campus of International Excellence (Andalucía Tech). The required sample size was calculated as 38 participants for the purposes of reliability testing, with an expected Cronbach α of 0.80 and an expected precision of 0.1 at 95% CI [28]. Given the projected rate of nonparticipation of 20%, the final sample size was determined to be 48.

#### Recruitment

Participants were recruited through direct invitations from the involved professors during class time. Those who agreed to take part in the study were required to complete a web-based informed consent form. Inclusion criteria were an absence of any known medical illness and ownership of a smartphone.

#### Setting

Participants performed the tasks on their smartphones and in their own classrooms and used a Google Form to submit their answers to the S-MAUQ.

#### Study Procedures

Participants were instructed to install the Yazio app on their mobile phones. Yazio, a standalone mHealth app for patients, was selected for the usability study due to its popularity. It is one of the most widely used nutrition apps in the world, helping people lead healthier lives globally. Its availability on both Android and iPhone devices [29,30] combined with its similarity to MyFitnessPal—an app frequently used in previous studies but that requires additional resources to be used—made it a suitable choice. A concise overview of the Yazio app, along with the essential steps for preliminary configuration, was displayed on the classroom projector. This procedure should be carried out exclusively at the point of the initial execution of the app following its installation.

Subsequently, participants were required to undertake the tasks detailed in Textbox 1.

Textbox 1. **Sheet of tasks to be performed using the Yazio app.**

1. Create initial profile when running the app for the first time (take a screenshot of the screen that appears at the end of the process, after discarding the tutorial)
2. Use the functions available in the Daily and/or Profile menu to add records or data for today:
  - Add the following foods consumed during breakfast
    - 1 black coffee
    - 2 slices of bread (bimbo) without crust
    - Natural orange juice, 200 mL
    - Olive oil, 1 tablespoon
    - Product with barcode 8410095040362 (or scan it from the product code photo)
  - Modify (in the profile) daily water amount goal: 2.5 liters
  - Add water consumed: 1.75 liters
  - Add activities (exercise completed) by pressing “More”
    - “walk, general” 30 minutes
    - “fast walk (6 km/h)” 10 minutes
  - Add 2 notes or indications about how your day is going
    - “I slept well”
    - “bored”
  - (Finally, take the necessary screenshots, 2 or 3, to collect the data entered)
3. Identify and note (via screenshot):
  - The number of calories consumed during the day (due to ingested foods and drinks)
  - Calories burned (from exercising, etc.)
  - Calories we have left to consume to reach the day’s goal
4. Explore and find a recipe that meets each of the following conditions:
  - 1 vegan breakfast (between 150 and 200 kcal) and mark it as a favorite
  - 1 vegan lunch (between 350 and 400 kcal) and mark it as a favorite
  - See the recipes you have marked as favorites (and take a screenshot of them)

First, it is recommended that an initial profile be created and the desired weight goals be determined. Second, to add records or data pertaining to the current day, the functions available from the Daily and/or Profile menus need to be used. Third, it is requested that the number of calories consumed throughout the day be identified and recorded. This should include the calories derived from food and drink, the calories expended through exercise, and the remaining calories to be consumed to reach the daily goal. Fourth, it is required to conduct a comprehensive investigation and identify a recipe that aligns with the specific criteria outlined in step 4 in Textbox 1.

Following the completion of the tasks, the participants were invited to respond to the S-MAUQ in person using a Google Form.

The internal consistency of the S-MAUQ was evaluated by calculating the Cronbach α value for the entire questionnaire and its 3 subscales. A higher α value indicates greater internal reliability, and a value of ≥0.70 is generally considered acceptable as an indication of good internal reliability [31,32]. All statistical analyses were conducted using SPSS (IBM Corp), and the results were verified using Excel (Microsoft Corp).

Figure 1 provides an overview of the process of translation and cross-cultural adaptation as well as the subsequent validation stage for S-MAUQ’.

### Ethical Considerations

No financial compensation was provided to participants at any stage of the study. Participation was voluntary and anonymous, except for members of the expert panel responsible for content validation, who were intentionally selected for their expertise. All participants (including the expert panel) were provided and signed an informed consent online, which included the possibility of opting out. The study adhered to the Declaration of Helsinki and was approved by the Bioethics Committee of the Medical University of Málaga (17‐2022-H).

## Results

### Content Validation

Please refer to Table 1, which presents the calculated values (using Excel) of the I-CVI for relevancy and clarity, the (Pc), and the modified kappa agreement (κ) by the 10 experts for each item of the S-MAUQ. The S-CVI was calculated to be 0.778. However, as can be seen, there were 2 items (#3 and #6) with κ<0.4 (interpreted as poor agreement) and 2 others (#15 and #18) with 0.4<κ<0.6 (interpreted as fair). Considering these findings, we sought to further refine the instrument in accordance with the recommendations of the expert panel.

**Table 1.** Content validity index of item relevancy and modified kappa agreement by 10 experts in round 1.

Items	Rating of 4 or 5, n	Rating of 1 to 3, n	Item-level content validity index^a^	Probability of chance occurrence^b^	Modified kappa^c^^,d^	Interpretation
1	10	0	1.000	0.001	1.000	Excellent
2	9	1	0.900	0.010	0.899	Excellent
3	5	5	0.500	0.246	0.337	Poor
4	8	2	0.800	0.044	0.791	Excellent
5	9	1	0.900	0.010	0.899	Excellent
6	5	5	0.500	0.246	0.337	Poor
7	10	0	1.000	0.001	1.000	Excellent
8	8	2	0.800	0.044	0.791	Excellent
9	7	3	0.700	0.117	0.660	Good
10	8	2	0.800	0.044	0.791	Excellent
11	9	1	0.900	0.010	0.899	Excellent
12	9	1	0.900	0.010	0.899	Excellent
13	8	2	0.800	0.044	0.791	Excellent
14	7	3	0.700	0.117	0.660	Good
15	6	4	0.600	0.205	0.497	Fair
16	9	1	0.900	0.010	0.899	Excellent
17	7	3	0.700	0.117	0.660	Good
18	6	4	0.600	0.205	0.497	Fair

a Scale content validity index=0.778.

b Computed using the formula Pc = [ N! / (A! * (N–A)!) ] * 0.5N, where Pc is the probability of chance occurrence, n is the number of experts, and A is the number of experts who agree the items are relevant/clear.

c Computed using the formula κ=(item-level content validity index – Pc) / (1 – Pc), where Pc is the probability of chance occurrence.

d Kappa was interpreted according to the following criteria: excellent, κ>0.74; good, 0.6≤κ≤0.74; fair, 0.40≤κ≤0.59; poor, κ<0.40.

The results of the second round are presented in Table 2, which indicates an S-CVI of 0.850. However, 2 items (#14 and #15) demonstrated a markedly low level of relevance and clarity (I-CVI), accompanied by a similarly low κ (0.4≤κ≤0.6, interpreted as “fair”). Following a thorough review with our panel of experts, it was determined that both items should be removed from the preliminary Spanish questionnaire (S-MAUQ). In conclusion, the S-CVI was calculated to be 0.881 (without considering the 2 items that were subsequently eliminated).

**Table 2.** Content validity index of item relevancy and modified kappa agreement by 10 experts in round 2.

Items	Rating of 4 or 5, n	Rating of 1 to 3, n	Item-level content validity index^a^	Probability of chance occurrence^b^	Modified kappa^c^^,^^d^	Interpretation
1	10	0	1.000	0.001	1.000	Excellent
2	9	1	0.900	0.010	0.899	Excellent
3	8	2	0.800	0.044	0.791	Excellent
4	8	2	0.800	0.044	0.791	Excellent
5	10	0	1.000	0.001	1.000	Excellent
6	10	0	1.000	0.001	1.000	Excellent
7	10	0	1.000	0.001	1.000	Excellent
8	8	2	0.800	0.044	0.791	Excellent
9	7	3	0.700	0.117	0.660	Good
10	9	1	0.900	0.010	0.899	Excellent
11	10	0	1.000	0.001	1.000	Excellent
12	10	0	1.000	0.001	1.000	Excellent
13	9	1	0.900	0.010	0.899	Excellent
14	6	4	0.600	0.205	0.497	Fair
15	6	4	0.600	0.205	0.497	Fair
16	8	2	0.800	0.044	0.791	Excellent
17	8	2	0.800	0.044	0.791	Excellent
18	7	3	0.700	0.117	0.660	Good

a Scale content validity index=0.850.

b Computed using the formula Pc = [ N! / (A! * (N–A)!) ] * 0.5N, where Pc is the probability of chance occurrence, n is the number of experts, and A is the number of experts who agree the items are relevant/clear.

c Computed using the formula κ=(item-level content validity index – Pc) / (1 – Pc), where Pc is the probability of chance occurrence.

d Kappa was interpreted according to the following criteria: excellent, κ>0.74; good, 0.6≤κ≤0.74; fair, 0.40≤κ≤0.59; poor, κ<0.40.

### Face Validation

Please refer to Table 3 for the calculated values of the I-FVI, the Pc, and the modified kappa agreement (κ) for each item. These were determined by the 12 target users. The S-FVI was calculated to be 0.927. A score >0.79 indicates that the items in the questionnaire are sufficiently clear and understandable for the nonexpert target user [27]. Furthermore, the modified kappa agreement for each item in S-MAUQ demonstrated excellent concordance (κ>0.74).

**Table 3.** Face validity index of item understandability and modified kappa agreement by 12 target users.

Items	Rating of 3 or 4, n	Rating of 1 or 2, n	Item-level face validity index^a^	Probability of chance occurrence^b^	Modified kappa^c^	Interpretation
1	12	0	1.000	0.000	1.000	Excellent
2	12	0	1.000	0.000	1.000	Excellent
3	9	3	0.750	0.054	0.740	Excellent
4	11	1	0.917	0.003	0.916	Excellent
5	11	1	0.917	0.003	0.916	Excellent
6	11	1	0.917	0.003	0.916	Excellent
7	11	1	0.917	0.003	0.916	Excellent
8	11	1	0.917	0.003	0.916	Excellent
9	10	2	0.833	0.016	0.831	Excellent
10	10	2	0.833	0.016	0.831	Excellent
11	12	0	1.000	0.000	1.000	Excellent
12	12	0	1.000	0.000	1.000	Excellent
13	12	0	1.000	0.000	1.000	Excellent
14^e^	—^f^	—	—	—	—	—
15^e^	—	—	—	—	—	—
16	12	0	1.000	0.000	1.000	Excellent
17	11	1	0.917	0.003	0.916	Excellent
18	11	1	0.917	0.003	0.916	Excellent

a Scale face validity index=0.927.

b Computed using the formula Pc = [ N! / (A! * (N–A)!) ] * 0.5N, where Pc is the probability of chance occurrence, n is the number of users, and A is the number of users who agree the items are understandable.

c Computed using the formula: =(item-level face validity index – Pc) / (1 – Pc), where Pc is the probability of chance occurrence.

d Kappa was interpreted according to the following criteria: excellent, κ>0.74; good, 0.6≤κ≤0.74; fair, 0.40≤κ≤0.59; poor, κ<0.40.

e Removed from the questionnaire.

f Not applicable.

### Reliability (Internal Consistency) Validation

A total of 61 participants completed the web-based consent form and the S-MAUQ questionnaire for the purposes of the reliability study (the calculated sample size was 48). The participants were between the ages of 18 years and 22 years, and all were students (41 men, 20 women). The mean usability score (normalized to 1) for the Yazio app, as determined by the S-MAUQ, was 0.855 (SD 0.088). This indicates a high level of usability.

The Cronbach α value for the entire S-MAUQ was 0.87, and the values for the 3 subscales were 0.74 for the ease of use dimension (Q1-Q5), 0.74 for the interface and satisfaction dimension (Q6-Q12), and 0.64 for the usefulness dimension (Q13-Q16). The new numbering scheme was used for Q13-Q16, omitting the 2 deleted items.

Furthermore, the Cronbach α value for the complete questionnaire remained highly consistent when an item was deleted (see Table 4), indicating that the S-MAUQ exhibited good internal reliability.

**Table 4.** The internal consistency of the item’s total statistics.

Items	Scale mean if item deleted^a^	Scale variance if item deleted	Corrected item total correlation	Cronbach α if item deleted^b^
1	89.46	87.086	0.532	0.854
2	89.07	85.129	0.635	0.850
3	89.10	85.190	0.570	0.852
4	89.07	87.562	0.470	0.857
5	89.15	84.061	0.462	0.859
6	88.90	89.323	0.345	0.863
7	89.49	87.754	0.439	0.858
8	88.97	89.432	0.403	0.860
9	89.13	87.949	0.436	0.858
10	89.28	85.404	0.593	0.851
11	89.74	79.797	0.599	0.851
12	89.13	85.949	0.702	0.849
13	89.20	88.294	0.442	0.858
14^c^	—^d^	—	—	—
15^c^	—	—	—	—
16	89.20	87.961	0.451	0.858
17	90.00	85.033	0.429	0.860
18	89.33	85.324	0.525	0.854

a Overall scale mean=95.21.

b Overall scale Cronbach α=0.87 (high internal consistency); ease of use subscale (Q1..Q5) Cronbach α=0.74; interface and satisfaction subscale (Q6..Q12) Cronbach α=0.74; usefulness subscale (Q13, Q16..Q18) Cronbach α=0.64.

c Removed from the questionnaire.

d Not appliclable.

## Discussion

### Principal Findings

Ensuring optimal usability of Spanish mobile apps is one of the most crucial factors for their use. Questionnaires are a common method for evaluating usability. This study outlined the process of translating, adapting, and validating the English version of the standalone MAUQ questionnaire for the patient into Spanish.

To this end, we followed the 4-step process proposed by Sousa and Rojjanasrirat [20], as it was outlined in the Methods section.

During the translation and adaptation phase, several meetings were held to ensure conceptual and linguistic equivalence for key terms such as interface, consistency, coherence, social setting, and health and well-being services. Final wording was established by consensus, often in consultation with the original questionnaire’s authors.

In the content validation phase, the expert panel initially did not reach full agreement on several items. A second round of review narrowed the discrepancies but left 2 items (#14 and #15) unresolved. Both items were judged to be conceptually ambiguous and difficult to interpret, even for experts. Following a series of email consultations with the original authors on the meaning of and convenience of these items and their overlap with others, we confirmed the experts’ results and their suggestions that these items could be removed from the final Spanish version.

During the face validation phase, the calculated FVI and κ coefficient yielded an S-FVI that substantially exceeded the recommended threshold, thereby ensuring the quality of the translation. The results of the study indicated that the items on the questionnaire were comprehensible and suitable for the target population.

Finally, the reliability analysis demonstrated strong internal consistency, with an overall Cronbach α of 0.87. The Spanish version maintained the original 3-dimensional structure of the English questionnaire, permitting direct cross-language comparison. Only the “usefulness” dimension was slightly affected, with an acceptable Cronbach α of 0.64 for exploratory studies. Overall, the S-MAUQ exhibited sound psychometric properties, indicating that it is a reliable and valid tool for assessing the usability of mobile health apps among Spanish-speaking patients. The final iteration of the S-MAUQ is presented in Table 5 for reference.

**Table 5.** The final Spanish version of the mHealth App Usability Questionnaire (S-MAUQ).

Item number	Question
1	La aplicación era fácil de usar.
2	Me resultó fácil aprender a utilizar la aplicación.
3	Al pasar de una pantalla a otra, la navegación era consistente, similar, previsible.
4	La aplicación me permitió usar todas las funciones que necesité (tales como visualizar e introducir información, responder a recordatorios, etc).
5	Cuando cometí un error usando la aplicación, fue fácil y rápido deshacerlo y volver a intentarlo.
6	Me gusta el diseño y aspecto de la aplicación.
7	La presentación de la información en la aplicación estaba bien organizada, y me permitió encontrar lo que buscaba.
8	La aplicación mostraba adecuadamente el progreso de mi actividad mientras interactuaba con ella.
9	Me sentiría cómodo/a utilizando esta aplicación en público, delante de otras personas.
10	El tiempo invertido en realizar las tareas deseadas me ha parecido adecuado.
11	Volvería a utilizar esta aplicación.
12	En general, estoy satisfecho/a con esta aplicación.
13	Creo que la aplicación puede ser útil para mi salud y bienestar.
14 (#16 in MAUQ)	Esta aplicación tiene todas las funciones que esperaba.
15 (#17 in MAUQ)	He podido realizar algunas tareas incluso cuando la conexión a internet era mala o no estaba disponible.
16 (#18 in MAUQ)	Esta aplicación me proporcionó una forma aceptable de recibir servicios de salud, tales como el acceso a material educativo, el seguimiento de mis actividades, o la realización de autoevaluaciones.

### Comparison With Prior Research

Our findings align with prior validations of the MAUQ conducted to assess a variety of mobile apps developed for different purposes and user groups, including the MyFitnessPal in Malaysia for evaluating caloric intake, weight goals, and nutritional guidance [16]; the Left-Handed Doctor mHealth app in China for medical consultations and public health guidance [33]; the Enable app in Germany for cancer patients to register electronic patient-reported outcomes and monitor treatment side effects [17]; and the DIGICOG-MS app in Italy for cognitive assessment of individuals with multiple sclerosis [18] and patients with osteoporosis in the Persian study [34]. Collectively, our findings suggest that both the MAUQ and S-MAUQ demonstrate stable content validity across diverse contexts and could be applied to a wide range of mHealth and eHealth applications designed for different populations.

It is noteworthy that a Spanish adaptation [19] developed in parallel with our own work has not adhered to the MAUQ standard; instead, it was adapted for breastfeeding. Several items have undergone alterations and been reordered, and one item was eliminated. The original dimensions were modified from 3 to 4, but the results indicate a significant lack of internal consistency in 2 final subscales. Furthermore, there was an erroneous interpretation and implementation of the formula applied to calculate the level of relevance and clarity of the questionnaire items. Consequently, it was not possible to achieve comparable outcomes in different languages.

We also compared our work with the aforementioned studies that focus on the “standalone, for patients” version of the MAUQ. The Malaysian version of the MAUQ [16] underwent an analysis similar to ours, maintaining the 3-dimensional structure but without eliminating any element. The German version of the MAUQ [17] also performed a study similar to ours and to that of the Malaysian version. However, the creators of the German version considered eliminating 1 item but finally decided not to do so in order to maintain consistency with the original. The Italian version [18] did not undergo content or face validation; they had a panel of 6 experts who directly compared a translated version with the original questionnaire and made modifications to have a more understandable Italian version.

### Limitations

In our study, as in other similar work [16], we surveyed a large sample of native Spanish-speaking students to provide a representative sample of the young adult demographic. In future research, criterion validation studies should be conducted to predict different outcomes for different domains and types of users of the S-MAUQ.

### Conclusions

We addressed a significant gap in the field of health app evaluation in Spanish by translating and adapting the MAUQ, one of the most recognized usability questionnaires in this area. Despite the need to eliminate 2 items due to their lack of relevance and demonstrated confusion, S-MAUQ is a reliable tool in Spanish for evaluating the usability of mHealth apps.

## Abbreviations

I-CVI: item-level content validity index
I-FVI: item-level face validity index
MARS: Mobile App Rating Scale
MAUQ: mHealth App Usability Questionnaire
mHealth: mobile health
Pc: probability of chance occurrence
S-CVI: scale content validity index
S-FVI: scale face validity index
S-MAUQ: Spanish version of the MAUQ
uMARS: MARS user version
